# Production of Oxidative and Hydrolytic Enzymes by *Coprinus cinereus* (Schaeff.) Gray from Sisal Wastes Supplemented with Cow Dung Manure

**DOI:** 10.1155/2015/650543

**Published:** 2015-11-19

**Authors:** Prosper Raymond, Anthony Manoni Mshandete, Amelia Kajumulo Kivaisi

**Affiliations:** Department of Molecular Biology and Biotechnology, College of Natural and Applied Sciences, University of Dar es Salaam, Uvumbuzi Road, P.O. Box 35179, Dar es Salaam, Tanzania

## Abstract

The activity of oxidative and hydrolytic enzymes of the edible and medicinal white rot fungi* Coprinus cinereus *(Schaeff.) Gray mushroom was observed during mycelia growth and fruiting body development in solid substrate fermentation using sisal waste fractions amended with cow dung manure as supplement. Laccase had the highest titre value among the five detected enzymes. Its activity was higher during mycelia growth compared to fruiting phase, with 10% supplemented substrate formulation unmixed sisal leaf decortication residues [abbreviated SL : SB (100 : 0)] displaying the highest activity of 39.45 ± 12.05 Ug^−1^. Lignin peroxidase (LiP) exhibited a characteristic wave-like pattern with the highest peaks found either during full mycelia colonization or soon after first flush harvest; the highest activity of 1.93 ± 0.62 Ug^−1^ was observed on unsupplemented SL : SB (100 : 0) substrate formulation during mycelia colonization. For hydrolytic enzymes, the highest carboxymethyl cellulase (CMCase) activity of 2.03 ± 0.70 Ug^−1^ was observed on 20% supplemented SL : SB (0 : 100) after first flush; that of pectinase (1.90 ± 0.32 Ug^−1^) was revealed after third flush on 10% supplemented SL : SB (0 : 100) substrate formulation while 10% supplemented SL : SB (25 : 75) exhibited the highest xylanase activity (1.23 ± 0.12 Ug^−1^) after first flush. These findings show that the activities of both oxidative and hydrolytic enzymes were regulated in line with developmental phase of growth of* Coprinus cinereus*.

## 1. Introduction

Enormous quantities of organic wastes from agricultural and food processing industries which are lignocellulosic in nature are generated annually [1]. A large proportion of these wastes are dumped or left to rot near the factories, burnt, or shredded and composted and used for landfill or improving soil quality [2]. However, with the application of appropriate bioconversion technology, these wastes represent a valuable resource especially for areas with agricultural-based economies.

Edible mushroom cultivation represents one of the most economically viable processes for the bioconversion of these lignocellulosic wastes which include diverse materials such as cereal straws, bagasse, sawdust, cotton wastes, banana leaves, coffee grounds, and sisal wastes [3–5]. Utilization of insoluble lignocellulosic substrates by these fungi depends on their capability to synthesize the relevant hydrolytic (cellulases and hemicellulases) and oxidative (ligninolytic) extracellular enzymes [6]. These are responsible for the degradation of substrate major components, that is, cellulose, hemicellulose, and lignin, into low molecular weight compounds that can be assimilated for fungal nutrition [7]. Production of these enzymes by the fungal mycelium is a crucial part of the colonization process and an important determinant of mushroom growth, development, and yields [8, 9]. Consequently, it is important to evaluate basidiomycetes hydrolytic and oxidative enzymes activity while cultivating in the presence of lignocellulose since in lignified plant substrates cellulose, hemicelluloses, and lignin are linked intra- and intermolecularly [6].

In Tanzania, high quantities of solid sisal wastes are produced annually as only 2% of the plants are currently used for sisal fibre production while the remaining 98% are commonly disposed of in the raw into the environment [10–12]. Although their use in the general cultivation of* Coprinus cinereus* has been established [4, 5], no information exists regarding enzymatic activity, function, and degradative capacity of* Coprinus* when cultivated upon this substrate. The aim of this work was, therefore, to determine the profile of the oxidative and hydrolytic enzymes during vegetative growth, fruiting, and postharvest stage in three flushes of* Coprinus cinereus* on solid sisal waste fractions supplemented with cow manure.

## 2. Material and Methods

### 2.1. Fungal Strain, Tissue Culture, and Spawn Preparation

*Coprinus cinereus* (Schaeff.) Gray was collected from sisal decortications waste dumpsite at Kidugalo sisal estate, Morogoro, Tanzania. These mushrooms were brought to the laboratory on the same day for tissue culture. The mycelia from living mushroom fruit bodies were aseptically obtained following the tissue culture protocol according to Dhouib et al. [13]. Spawn were prepared with intact sorghum grains bought from Kariakoo market, Dar es Salaam, according to Mshandete and Cuff [4].

### 2.2. Substrate Preparation and Mushroom Cultivation Experiments

Fresh sisal leaf decortication residues, a leafy biomass produced during sisal decortications and sisal boles, were obtained from Kidugalo sisal decortications factory at Morogoro region, Tanzania. These substrates were further pretreated prior to* Coprinus cinereus* cultivation as reported in Raymond et al. [5]. The substrates for mushroom cultivation consisted of composted sisal leaf (SL) decortication residues and sisal boles (SB) formulated in proportions of (100 : 0), (75 : 25), (50 : 50), (25 : 75), and (0 : 100) and supplemented with cow manure at the rate of 10%, 20%, and 30% on dry weight basis. Cultivation was carried out in solid state fermentation bioreactors (SSFBs) which consisted of 3-litre rectangular plastic containers with dimension of 23 cm × 14 cm × 9 cm (length, width, and height, resp.) (Cello Domestic Ware (Mkate), Dar es Salaam, Tanzania). A total of 136 aeration holes of 0.7 cm in diameter and 3 cm apart were made in all the sides of the bioreactor to facilitate aeration during spawn running. All substrates were inoculated with fully colonized 14-day old spawn at 6% (wet weight spawn/wet weight substrate) under aseptic conditions. The conditions during spawn running in the room were 28 ± 2°C and relative air humidity of 78 ± 2%. Once the mycelia of* C. cinereus* strain had grown throughout the whole substrate, the bioreactors were removed and transferred to a fruiting room, where the environment was illuminated by sunlight. Relative humidity in the room was increased to 86 ± 4% while temperature was decreased to 26.5 ± 0.05°C, respectively, by pouring 25 litres of cold water and ice cubes per day on the floor. The mushroom fruiting bodies were harvested according to Mshandete and Cuff [4].

### 2.3. Extraction of Crude Enzymes from Substrates

Substrate samples with active mycelia of about 20 g (±2 g) were taken from the container at different intervals starting on the 5th day, 10th day (full colonization), 15th day (first flush), 20th day (second flush), and 25th day (third flush). These samples were weighed and mixed with 50 mL of the subsequent extraction buffer and homogenized. The mixture was agitated on orbital shaker (Edmund Bühler, 7400 Tübingen, Germany) for one hour at 120 rpm. During this step, all flasks containing the substrate-buffer mixture were kept on ice throughout the entire shaking period. These mixtures were then filtered through double layered cheesecloth followed by centrifugation at 6000 rpm for 10 minutes (Mikro 22R centrifuge, Hettich, England) at 4°C to remove residual particles. The clear supernatants obtained were regarded as crude enzymes which were later used for enzyme activity assays.

### 2.4. Enzyme Assays

Laccase production in the crude enzyme extract was determined according to Risdianto et al. [14] by monitoring the absorbance change at 420 nm (*A*_420 nm_) related to the rate of oxidation of 1 mM 2,2′-azino-bis-[3-ethylbenzthiazoline-6-sulfonate] (ABTS) in 50 mM sodium acetate buffer (pH 5.5). Assays were performed in 1.5 mL cuvette containing 0.5 mM of ABTS in 50 mM sodium acetate buffer (pH 5.5) and 50 *μ*L of crude enzyme [15]. The oxidation of ABTS was monitored by the formation of intense blue-green color which was observed by measuring the increase in the absorbance at 420 nm, with a molar extinction coefficient of 36,000 M^−1 ^cm^−1^ using UV-Visible spectrophotometer (Jenway, Genova, Bibby Scientific Ltd., England). One unit of activity was defined as the amount of enzyme which leads to the oxidation of 1 *μ*mol of ABTS per minute.

Lignin peroxidase activity was determined at 30°C using the method of Sugiura et al. [16] in a reaction mixture (1.5 mL) containing 20 mM citrate buffer (pH 3.0), 10 mM veratryl alcohol (= 3,4-dimethoxybenzyl alcohol), and 0.1 mL crude enzyme extract. The reaction was started by adding 10 mM H_2_O_2_ in the assay mixture after incubation at 30°C for 5 minutes, and the increase in absorbance was followed spectrophotometrically using Jenway UV-Visible spectrophotometer (Bibby Scientific Ltd., United Kingdom) at 310 nm (extinction coefficient, *ε*310 = 9300 M^−1 ^cm^−1^,) due to oxidation of veratryl alcohol to veratraldehyde (= 3,4-dimethoxybenzaldehyde). One unit (1 U) of LiP activity was defined as the amount of enzyme that oxidized 1.0 *μ*mole of veratryl alcohol per minute at pH 3 and 30°C.

Carboxymethyl cellulase (CMCase), pectinase, and xylanase activities were estimated using the DNS method according to Miller [17], measuring the amount of reducing sugars (as glucose for CMCase/pectinase or xylose in case of xylanase) liberated in reaction mixtures containing 0.5% carboxymethyl cellulose (Sigma), 0.5% pectin (citrus pectin, Sigma), or 0.5% birch wood xylan (Sigma) and supernatant, respectively. The incubation times were 30 and 60 minutes at 30°C, respectively [18]. Standard curves were obtained using glucose or xylose. Enzyme activities (U) were expressed as the amount of enzyme releasing 1 *μ*mole of glucose or xylose equivalent per mL per min. All enzyme assays were carried out in triplicate.

## 3. Results

### 3.1. Spawn Run and Pinhead and Fruiting Bodies Formation

The first well visible signs of fungal growth in solid state fermentation were seen two days after inoculation with* Coprinus cinereus* while the total vegetative growth (spawn running) on solid sisal waste fractions took an average of about 10 ± 2 days regardless of the supplementation rate used. The pinheads (primordia) appeared after 12 ± 3 days while it took 1-2 days for mature mushrooms to be ready for harvesting (Table 1). Three flushes of fruiting were obtained and the fruiting bodies in the first flushes weighed more than the fruiting bodies in subsequent flushes. This study revealed that different substrates showed varying yields and biological efficiencies with or without supplements. Results regarding mushroom yields, mushroom size, and biological efficiencies of these substrate formulations are reported elsewhere [5].

### 3.2. Enzyme Profile during Solid State Cultivation of* Coprinus cinereus* on Sisal Waste Formulations

#### 3.2.1. Laccase Activities (Ug^−1^ SMS Wet Wt.)

Results of laccase activity profiles during solid state cultivation of* C. cinereus* on various solid sisal waste formulations supplemented with cow manure are presented in Table 2. Activity levels in all substrate formulations were found to increase during vegetative growth phase up to when the substrates were full colonized (day 10), with 10% supplemented substrate formulation SL : SB (100 : 0) displaying the highest activity peak of 39.45 ± 12.05 Ug^−1^ at this stage. However, there was abrupt decline of these activities soon after the first harvest in almost all substrate formulations except for some on which activities were found to increase again after the third flush harvest.

#### 3.2.2. Lignin Peroxidase (Ug^−1^ SMS Wet Wt.)

The lignin peroxidase (LiP) activities from early colonization till fruiting bodies development were fluctuating with a wave-like pattern (Table 3). The peak activity occurred twice between the full mycelia colonization and the third flush harvest. The highest peaks were found to be either during full mycelia colonization or just after first flush harvest. The highest activity of 1.93 ± 0.62 Ug^−1^ was observed on unsupplemented SL : SB (100 : 0) substrate formulation during full mycelia colonization.

#### 3.2.3. CMCase Profile (Ug^−1^ SMS Wet Wt.)

The data in Table 4 indicate that the* C. cinereus* CMCase activity varied with different substrate formulation used. Higher activities were detected either during full mycelia colonization or soon after first flush harvest followed by gradual decline in the subsequent flushes. The highest activity of 2.03 ± 0.70 Ug^−1^ was observed on 20% supplemented SL : SB (0 : 100) after the first flush harvest.

#### 3.2.4. Pectinase (Ug^−1^ SMS Wet Wt.)

The pectinase activities were observed to increase gradually over days of cultivation (Table 5). Higher activities were found on spent substrates of either second or third flush. The highest pectinase activity of 1.90 ± 0.32 Ug^−1^ was revealed after the third flush harvest on 10% supplemented SL : SB (0 : 100) substrate formulation.

#### 3.2.5. Xylanase Enzymes (Ug^−1^ SMS Wet Wt.)

The xylanase activity varied with substrate formulation and supplementation rate during developmental stages. The results showed that xylanase activity was increasing as incubation days increase. It was found to be higher either during full mycelia colonization or after the first flush harvest; besides, the rate of xylanase production was slightly increased in fruiting phase compared with mycelia growth period. The highest activity of 1.23 ± 0.12 Ug^−1^ was observed on 10% supplemented SL : SB (25 : 75) after the first flush harvest (Table 6).

## 4. Discussion

### 4.1. Spawn Run and Pinhead and Fruiting Bodies Formation

The present study revealed that the efficient colonization and utilization by* C. cinereus* varied among solid sisal waste formulations with or without addition of supplement, which depended upon the ability of this fungus to produce extracellular enzymes required to degrade major components of the sisal waste biomass. It means that* C. cinereus* are capable of degrading lignocellulosic polysaccharides to ensure mycelia with carbon and energy source for extensive substrate colonization and fruit body formation [1]. In addition, not only the enzymes profiles but also their physicochemical characteristics when they are secreted account for the capacity of colonization and degradation; more stable enzymes at their optimum pH activity are capable of producing more extensive degradation of the substrate [19]. The number of days taken for completion of spawn run and those for pinheads formation differed significantly (*p* < 0.05) indicating the difference in composition among the substrate formulations with or without supplement. These results are in agreement with previously reported findings. Gaitán-Hernández et al. [20] did correlation studies between the constituents of the substrates and the number of days to primordium formation; they discovered a significant positive relationship with cellulose content and total sugar indicating that cellulose and sugar content in each substrate were directly proportional to the spawning duration and the primordium formation. Similarly, Oei [21] reported that substrate having high quality lignin and cellulose contents takes a longer time to start pinning and fruit body formation.

### 4.2. Enzyme Profile during Solid State Cultivation of* Coprinus cinereus* on Sisal Waste Formulations

Many fungal species have the ability to degrade lignocellulosic biomass by producing extracellular enzymes. The extracellular enzymes production by solid state fermentation plays a crucial role supplying carbon and energy source consequently providing the fungus with the material for biosynthetic activity. The present study focuses on measuring two oxidative (laccase and lignin peroxidase) and three hydrolytic (carboxymethyl cellulase, pectinase, and xylanase) enzymes secreted by* Coprinus cinereus*, using sisal waste fractions amended with cow manure. In addition, the study shows the profiles of extracellular enzymatic activities produced during different cultivation stages varied among the sisal waste substrate formulations supplemented with cow manure. The differences found in enzyme production might be due to the relative composition of polysaccharides, the size of the wastes used, and probably the presence of natural inducers such as aromatic compounds [22]. In general, the yields of lignocellulolytic enzymes were expected to increase as supplementation rate increases but this was not the case in this study. This could be due to repression effect caused by addition of cow manure [23]. Moreover, in the literature, contradictory evidence exists for the effects of the nature and concentration of the nitrogen source on ligninolytic enzyme production. While high nitrogen media gave the highest laccase activity in* Lentinus edodes*,* Rigidoporus lignosus*, and* Trametes pubescens*, nitrogen-limited conditions enhanced the production of the enzyme in* Pycnoporus cinnabarinus*,* P. sanguineus*, and* Phlebia radiata* [24, 25]. The role of these compounds in the regulation of enzyme synthesis depends not only on the physiology of the tested fungi but also on the medium composition, especially on the presence of lignocellulosic substrate [26, 27]. Of the lignocellulolytic enzymes assayed, laccase was detected in the highest quantity, in terms of total units per substrate.

Laccase was actively secreted during mycelia run and reached the maximum level during full mycelia colonization and then declined gradually during fruiting. The laccase increased again after harvesting either first or second flushes while in lignin peroxidase (LiP) a wave-like pattern was observed, with the highest peaks found during full mycelia colonization or soon after first flush harvest. Higher levels of laccase activity during early stage of the spawn run indicate that this enzyme is responsible for degradation of physical barrier of lignin so that more cellulosic and hemicellulosic content is exposed for further degradation to extract the energy for the growth of mushroom fungus [28]. It has also been well documented that composition of the substrate and the method of cultivation influence the pattern of enzyme production by white rot fungi [29, 30]. The present findings are similar as reported by several authors working with different basidiomycetes [30–33]. The study of Periasamy [34] using* Pleurotus djamor *var.* roseus* correlated the increase of laccase activity to growth and differentiation of fruiting bodies and decreasing levels of lignin content. Moreover, Thurston [35] showed that white rot fungi laccase, besides functioning as the lignin degrading enzyme, was also important in pigment production, polyphenol detoxification, fruiting body formation, and sporulation and as antimicrobial agent. In general, ligninolytic enzyme increased at somatic phase and decreased at the fruiting body phase. However, it is assumed that when the fruiting body was reformed at the next incubation, the fluctuation of ligninolytic enzyme activity occurred.

With respect to hydrolytic enzymes, CMCase enzyme activity gradually increased coinciding with fungal growth and maximum peaks were detected after either first flush or second flush (Table 4), suggesting a role in the morphogenesis of fruiting bodies as well as a function in overall nutrition of this mushroom species [36]. Sharma and Arora [37] associated elevated CMCase levels produced by mycelia on lignocellulosic substrates with high biological efficiencies.

Pectinase activity showed a gradual increase during mycelial growth and a sharp increase during fructification in all substrate formulations. A common feature among pectic enzymes that could be responsible for this increase of pectinase activity is their glucose repressible synthesis [38]. The results of xylanase production show a positive correlation with those obtained by Isikhuemhen and Mikiashvilli [39], who observed higher xylanase productivity from* Pleurotus ostreatus* in fruiting than mycelial growth period when cultivated on some solid wastes. Similarly, Terashita et al. [40] observed xylanase activity increasing during vegetative mycelial growth and maximum activity was reached after cropping of* P. flabellatus* and* P. sajor-caju*. The low activity of xylanase during the beginning of incubation could probably be due to the fact that xylanase was repressed by the ligninolytic enzyme, which was active in the beginning as reported by Singh et al. [28]. The increase in xylanase activity beyond full mycelia colonization is an indication that the enzyme was active in hydrolyzing hemicellulose during this stage. This also suggests that hemicellulose is not utilized in the beginning by the growing mycelium. However, a contrary result was found by Chen et al. [41] who reported that xylanolytic activity was crucial for the vegetative growth of fungus. Ahlawat et al. [42] also observed that xylanase was at peak on the 10th day during cultivation of* Volvariella volvacea* on paddy straw and was crucial during vegetative growth of the fungus.

## 5. Conclusion

In the present work, it was shown that* Coprinus cinereus* can be successfully cultivated on solid sisal waste formulations amended with cow dung manure probably because they produce enzymatic activities that are necessary for lignocellulose degradation, as well as for developmental regulation. The pattern of both hydrolytic and oxidative activity of* C. cinereus* enzymes was probably affected by the solid sisal waste substrates, growth stage, and the development of mushroom. The investigation of the hydrolytic and oxidative enzymes production during edible mushroom cultivation is required because after harvesting of fruit bodies the residual spent substrate may become a cheap source of lignocellulolytic enzymes for several applications including bioremediation and enzymes production as shown above.

## Figures and Tables

**Table 1:** Days for completion of spawn running and pinheads and fruiting bodies formation.

Supplement level	Substrate formulation	Days for completion of spawn run	Days for pinheads formation	Days for fruiting bodies formation
0%	SL : SB (100 : 0)	12 ± 1	15 ± 1	17 ± 1
	SL : SB (75 : 25)	11 ± 1	13 ± 1	15 ± 3
	SL : SB (50 : 50)	10 ± 1	11 ± 1	13 ± 2
	SL : SB (25 : 75)	10 ± 1	12 ± 1	13 ± 1
	SL : SB (0 : 100)	13 ± 2	14 ± 1	16 ± 1

10%	SL : SB (100 : 0)	11 ± 1	13 ± 1	15 ± 2
	SL : SB (75 : 25)	11 ± 1	14 ± 1	15 ± 1
	SL : SB (50 : 50)	10 ± 1	12 ± 1	13 ± 1
	SL : SB (25 : 75)	9 ± 1	10 ± 1	12 ± 2
	SL : SB (0 : 100)	12 ± 1	13 ± 1	14 ± 1

20%	SL : SB (100 : 0)	11 ± 1	14 ± 1	15 ± 1
	SL : SB (75 : 25)	10 ± 1	15 ± 1	17 ± 2
	SL : SB (50 : 50)	8 ± 1	10 ± 1	11 ± 1
	SL : SB (25 : 75)	9 ± 1	11 ± 1	12 ± 1
	SL : SB (0 : 100)	11 ± 1	12 ± 1	14 ± 1

30%	SL : SB (100 : 0)	10 ± 1	12 ± 0	13 ± 1
	SL : SB (75 : 25)	9 ± 1	12 ± 1	13 ± 1
	SL : SB (50 : 50)	10 ± 1	12 ± 1	13 ± 2
	SL : SB (25 : 75)	8 ± 1	9 ± 1	11 ± 1
	SL : SB (0 : 100)	12 ± 1	14 ± 1	15 ± 1

**Table 2:** Laccase activity (Ug^−1^ Spent Mushroom Substrate (SMS) wet weight) profiles of *Coprinus cinereus *at different stages during cultivation on solid sisal wastes supplemented with cow dung manure.

Supplement level	Days of incubation	Substrate formulations				
		SL : SB (100 : 0)	SL : SB (25 : 75)	SL : SB (50 : 50)	SL : SB (75 : 25)	SL : SB (0 : 100)
0%	5	6.87 ± 1.85	9.56 ± 2.85	8.29 ± 2.05	5.09 ± 1.23	11.04 ± 1.05
	10	34.87 ± 5.73	27.89 ± 10.56	20.05 ± 4.90	26.97 ± 8.92	24.67 ± 6.43
	15	18.06 ± 7.95	24.95 ± 7.50	23.93 ± 3.17	24.12 ± 1.03	17.23 ± 4.19
	20	15.82 ± 2.20	12.05 ± 2.19	13.89 ± 1.94	19.37 ± 3.15	6.52 ± 1.73
	25	27.68 ± 5.50	2.97 ± 1.12	9.56 ± 1.06	2.98 ± 0.87	13.98 ± 2.11

10%	5	3.09 ± 1.02	7.85 ± 1.98	9.86 ± 3.11	11.93 ± 3.13	6.93 ± 2.12
	10	39.45 ± 12.05	29.73 ± 4.83	26.17 ± 1.85	29.28 ± 7.93	26.58 ± 10.45
	15	28.89 ± 8.72	20.67 ± 3.51	17.83 ± 2.54	26.91 ± 4.82	24.91 ± 3.19
	20	10.34 ± 2.87	16.84 ± 4.97	4.04 ± 1.13	22.36 ± 3.12	17.48 ± 1.03
	25	8.55 ± 3.89	4.86 ± 2.67	12.14 ± 2.19	7.05 ± 4.92	9.87 ± 2.10

20%	5	12.04 ± 2.87	9.69 ± 2.65	10.73 ± 1.32	7.93 ± 1.49	14.93 ± 3.01
	10	28.67 ± 10.55	25.09 ± 1.86	27.85 ± 7.57	27.38 ± 6.74	28.27 ± 8.83
	15	20.59 ± 8.97	19.76 ± 4.76	24.17 ± 4.98	21.99 ± 1.33	25.92 ± 2.10
	20	12.64 ± 4.89	11.98 ± 3.57	14.04 ± 3.01	12.04 ± 2.21	17.11 ± 2.18
	25	16.54 ± 1.59	14.09 ± 2.95	18.61 ± 2.82	6.92 ± 1.04	7.98 ± 1.13

30%	5	8.99 ± 2.07	5.82 ± 1.64	12.09 ± 3.18	6.16 ± 2.14	8.95 ± 2.03
	10	30.08 ± 3.03	28.76 ± 3.54	32.96 ± 12.75	31.85 ± 11.85	29.74 ± 9.47
	15	25.98 ± 6.73	17.67 ± 3.64	26.82 ± 3.92	25.91 ± 3.10	21.81 ± 0.99
	20	18.30 ± 7.54	14.89 ± 3.76	3.59 ± 0.91	18.73 ± 1.99	10.83 ± 3.19
	25	9.45 ± 3.09	5.91 ± 1.65	19.73 ± 5.03	9.74 ± 2.18	15.98 ± 1.96

**Table 3:** Lignin peroxidase (LiP) activity (Ug^−1^ SMS wet wt.) profiles of *Coprinus cinereus* at different stages during cultivation on solid sisal wastes supplemented with cow dung manure.

Supplement level	Days of incubation	Substrate formulations				
		SL : SB (100 : 0)	SL : SB (25 : 75)	SL : SB (50 : 50)	SL : SB (75 : 25)	SL : SB (0 : 100)
0%	5	0.59 ± 0.13	0.34 ± 0.09	0.53 ± 0.23	0.27 ± 0.11	0.37 ± 0.13
	10	1.93 ± 0.62	0.98 ± 0.21	1.48 ± 0.17	0.89 ± 0.10	0.67 ± 0.22
	15	0.78 ± 0.20	1.07 ± 0.30	1.07 ± 0.28	1.06 ± 0.27	0.89 ± 0.17
	20	0.91 ± 0.11	0.72 ± 0.17	0.34 ± 0.10	0.45 ± 0.19	0.53 ± 0.10
	25	0.43 ± 0.18	0.27 ± 0.12	0.41 ± 0.08	0.53 ± 0.21	0.35 ± 0.14

10%	5	0.68 ± 0.24	0.49 ± 0.15	0.34 ± 0.07	0.58 ± 0.22	0.47 ± 0.12
	10	1.21 ± 0.40	1.04 ± 0.28	1.32 ± 0.34	1.10 ± 0.43	0.84 ± 0.17
	15	0.99 ± 0.19	0.65 ± 0.18	1.01 ± 0.23	0.98 ± 0.29	0.61 ± 0.20
	20	1.04 ± 0.30	0.74 ± 0.20	0.67 ± 0.17	0.49 ± 0.10	0.73 ± 0.09
	25	0.67 ± 0.15	0.41 ± 0.12	0.48 ± 0.12	0.31 ± 0.07	0.46 ± 0.17

20%	5	0.48 ± 0.16	0.57 ± 0.24	0.41 ± 0.10	0.42 ± 0.06	0.53 ± 0.14
	10	1.09 ± 0.20	1.17 ± 0.47	1.12 ± 0.33	0.85 ± 0.15	1.04 ± 0.30
	15	0.85 ± 0.34	0.87 ± 0.23	0.78 ± 0.14	1.03 ± 0.43	0.67 ± 0.21
	20	0.97 ± 0.26	0.93 ± 0.10	0.85 ± 0.25	0.67 ± 0.11	0.71 ± 0.13
	25	0.38 ± 0.10	0.27 ± 0.14	0.53 ± 0.12	0.46 ± 0.18	0.56 ± 0.18

30%	5	0.61 ± 0.12	0.53 ± 0.17	0.44 ± 0.16	0.50 ± 0.20	0.40 ± 0.14
	10	0.89 ± 0.19	1.07 ± 0.43	1.64 ± 0.48	1.13 ± 0.38	1.22 ± 0.20
	15	1.09 ± 0.32	0.81 ± 0.32	0.89 ± 0.29	0.74 ± 0.13	0.78 ± 0.11
	20	0.72 ± 0.29	0.56 ± 0.11	0.97 ± 0.19	0.87 ± 0.10	0.81 ± 0.29
	25	0.49 ± 0.14	0.32 ± 0.10	0.57 ± 0.13	0.49 ± 0.17	0.36 ± 0.10

**Table 4:** Carboxymethyl cellulase (CMCase) activity (Ug^−1^ SMS wet wt.) profiles of *Coprinus cinereus* at different stages during cultivation on solid sisal wastes supplemented with cow dung manure.

Supplement level	Days of incubation	Substrate formulations				
		SL : SB (100 : 0)	SL : SB (25 : 75)	SL : SB (50 : 50)	SL : SB (75 : 25)	SL : SB (0 : 100)
0%	5	0.67 ± 0.07	0.47 ± 0.12	0.19 ± 0.08	0.26 ± 0.08	0.39 ± 0.10
	10	1.29 ± 0.13	0.35 ± 0.12	0.49 ± 0.10	0.40 ± 0.13	1.02 ± 0.40
	15	0.80 ± 0.03	0.79 ± 0.28	0.71 ± 0.15	0.81 ± 0.21	1.35 ± 0.08
	20	0.36 ± 0.10	0.95 ± 0.20	0.40 ± 0.09	1.48 ± 0.40	0.68 ± 0.20
	25	0.26 ± 0.09	0.19 ± 0.06	0.55 ± 0.15	0.29 ± 0.09	0.34 ± 0.11

10%	5	0.34 ± 0.10	0.44 ± 0.10	0.24 ± 0.08	0.33 ± 0.12	0.47 ± 0.15
	10	0.92 ± 0.20	0.59 ± 0.20	0.58 ± 0.18	0.67 ± 0.25	0.45 ± 0.10
	15	1.09 ± 0.09	0.78 ± 0.13	0.98 ± 0.20	0.91 ± 0.04	1.67 ± 0.20
	20	0.27 ± 0.05	0.40 ± 0.12	0.32 ± 0.10	0.34 ± 0.07	1.23 ± 0.30
	25	0.22 ± 0.12	0.18 ± 0.06	0.51 ± 0.14	0.28 ± 0.12	0.48 ± 0.09

20%	5	0.29 ± 0.08	0.59 ± 0.17	0.35 ± 0.10	0.48 ± 0.20	0.41 ± 0.20
	10	0.88 ± 0.03	0.75 ± 0.05	0.50 ± 0.09	0.35 ± 0.10	1.53 ± 0.30
	15	0.93 ± 0.28	1.17 ± 0.30	0.86 ± 0.11	0.96 ± 0.12	2.03 ± 0.70
	20	0.62 ± 0.19	0.30 ± 0.09	0.33 ± 0.12	1.65 ± 0.30	0.57 ± 0.15
	25	0.18 ± 0.07	0.32 ± 0.10	0.17 ± 0.02	0.24 ± 0.06	0.36 ± 0.10

30%	5	0.64 ± 0.23	0.39 ± 0.08	0.20 ± 0.09	0.56 ± 0.15	0.37 ± 0.12
	10	0.72 ± 0.05	0.72 ± 0.12	0.69 ± 0.26	1.43 ± 0.30	0.97 ± 0.20
	15	1.37 ± 0.47	1.04 ± 0.20	0.48 ± 0.02	2.83 ± 1.12	1.13 ± 0.40
	20	1.06 ± 0.30	0.20 ± 0.04	0.10 ± 0.03	0.42 ± 0.07	0.68 ± 0.20
	25	0.49 ± 0.07	0.28 ± 0.09	0.45 ± 0.11	0.22 ± 0.09	0.16 ± 0.06

**Table 5:** Pectinase activity (Ug^−1^ SMS wet wt.) profiles of *Coprinus cinereus* at different stages during cultivation on solid sisal wastes supplemented with cow dung manure.

Supplement level	Days of incubation	Substrate formulations				
		SL : SB (100 : 0)	SL : SB (25 : 75)	SL : SB (50 : 50)	SL : SB (75 : 25)	SL : SB (0 : 100)
0%	5	0.13 ± 0.05	0.08 ± 0.03	0.19 ± 0.08	0.25 ± 0.10	0.36 ± 0.10
	10	0.36 ± 0.11	0.26 ± 0.12	0.13 ± 0.05	0.59 ± 0.18	0.49 ± 0.14
	15	0.76 ± 0.14	0.50 ± 0.12	0.36 ± 0.08	0.88 ± 0.10	1.16 ± 0.15
	20	0.97 ± 0.20	0.95 ± 0.11	0.60 ± 0.15	0.87 ± 0.22	0.46 ± 0.10
	25	1.46 ± 0.13	0.84 ± 0.05	0.79 ± 0.28	1.59 ± 0.40	1.12 ± 0.27

10%	5	0.23 ± 0.08	0.13 ± 0.05	0.11 ± 0.04	0.19 ± 0.09	0.27 ± 0.12
	10	0.74 ± 0.30	0.37 ± 0.13	0.26 ± 0.10	0.47 ± 0.16	0.51 ± 0.20
	15	1.32 ± 0.13	0.56 ± 0.10	0.47 ± 0.18	1.28 ± 0.21	1.32 ± 0.22
	20	1.05 ± 0.24	0.72 ± 0.14	0.86 ± 0.10	0.66 ± 0.09	0.28 ± 0.08
	25	1.90 ± 0.32	0.79 ± 0.15	0.59 ± 0.16	0.91 ± 0.17	0.57 ± 0.18

20%	5	0.18 ± 0.07	0.22 ± 0.09	0.18 ± 0.07	0.09 ± 0.02	0.19 ± 0.09
	10	0.57 ± 0.20	0.47 ± 0.14	0.31 ± 0.10	0.57 ± 0.12	0.47 ± 0.16
	15	1.10 ± 0.23	0.59 ± 0.17	0.59 ± 0.14	1.52 ± 0.37	1.81 ± 0.14
	20	0.71 ± 0.10	0.31 ± 0.07	0.77 ± 0.18	0.74 ± 0.10	0.38 ± 0.12
	25	0.22 ± 0.08	1.43 ± 0.18	0.99 ± 0.30	1.08 ± 0.30	0.64 ± 0.10

30%	5	0.21 ± 0.08	0.27 ± 0.11	0.20 ± 0.09	0.31 ± 0.14	0.29 ± 0.12
	10	0.75 ± 0.30	0.39 ± 0.10	0.25 ± 0.13	0.53 ± 0.20	0.68 ± 0.20
	15	1.22 ± 0.16	0.92 ± 0.03	0.42 ± 0.08	1.12 ± 0.18	1.77 ± 0.11
	20	0.98 ± 0.20	0.66 ± 0.20	0.84 ± 0.12	0.63 ± 0.04	0.63 ± 0.10
	25	1.79 ± 0.50	1.51 ± 0.11	0.58 ± 0.15	1.16 ± 0.28	0.40 ± 0.07

**Table 6:** Xylanase activity (Ug^−1^ SMS wet wt.) profiles of *Coprinus cinereus* at different stages during cultivation on solid sisal wastes supplemented with cow dung manure.

Supplement level	Days of incubation	Substrate formulations				
		SL : SB (100 : 0)	SL : SB (25 : 75)	SL : SB (50 : 50)	SL : SB (75 : 25)	SL : SB (0 : 100)
0%	5	0.09 ± 0.04	0.27 ± 0.09	0.17 ± 0.09	0.24 ± 0.10	0.15 ± 0.07
	10	0.58 ± 0.10	0.45 ± 0.14	0.37 ± 0.14	0.44 ± 0.14	0.33 ± 0.11
	15	0.79 ± 0.20	0.76 ± 0.12	0.97 ± 0.10	0.73 ± 0.12	1.06 ± 0.24
	20	0.21 ± 0.09	0.61 ± 0.10	0.87 ± 0.30	0.59 ± 0.06	0.66 ± 0.16
	25	0.15 ± 0.06	0.30 ± 0.18	0.41 ± 0.07	0.51 ± 0.09	0.31 ± 0.12

10%	5	0.15 ± 0.06	0.31 ± 0.07	0.27 ± 0.11	0.26 ± 0.08	0.18 ± 0.07
	10	0.27 ± 0.11	0.63 ± 0.20	0.43 ± 0.13	0.33 ± 0.10	0.24 ± 0.09
	15	0.39 ± 0.06	**1.23 ± 0.12**	0.81 ± 0.07	0.98 ± 0.14	0.70 ± 0.20
	20	0.18 ± 0.07	0.62 ± 0.09	0.96 ± 0.20	0.60 ± 0.15	0.48 ± 0.14
	25	0.10 ± 0.04	0.47 ± 0.10	0.56 ± 0.06	0.25 ± 0.07	0.33 ± 0.10

20%	5	0.24 ± 0.09	0.43 ± 0.10	0.16 ± 0.05	0.19 ± 0.07	0.15 ± 0.06
	10	0.67 ± 0.12	0.72 ± 0.22	1.09 ± 0.04	0.27 ± 0.11	0.50 ± 0.18
	15	0.95 ± 0.20	0.97 ± 0.18	0.92 ± 0.16	0.79 ± 0.15	0.89 ± 0.10
	20	0.39 ± 0.06	0.66 ± 0.09	0.49 ± 0.09	0.61 ± 0.09	0.55 ± 0.19
	25	0.14 ± 0.05	0.44 ± 0.14	0.42 ± 0.12	0.40 ± 0.07	0.16 ± 0.04

30%	5	0.39 ± 0.10	0.51 ± 0.16	0.23 ± 0.09	0.30 ± 0.10	0.19 ± 0.08
	10	0.47 ± 0.13	0.67 ± 0.23	0.36 ± 0.14	0.58 ± 0.19	0.32 ± 0.10
	15	1.04 ± 0.20	0.99 ± 0.07	0.94 ± 0.20	0.89 ± 0.08	0.88 ± 0.13
	20	0.76 ± 0.16	0.70 ± 0.15	0.63 ± 0.15	0.45 ± 0.11	0.45 ± 0.15
	25	0.31 ± 0.07	0.25 ± 0.12	0.41 ± 0.08	0.23 ± 0.07	0.36 ± 0.18
